# Incidental diagnosis of cardiac fibroma in an infant with acute viral bronchiolitis

**DOI:** 10.1590/1984-0462/2024/42/2022157

**Published:** 2024-05-27

**Authors:** Yasmine Gorczevski Pigosso, Izabela Mara Fogiato, Gabriela Guimarães Vieira, André Vaz, Ana Paula Percicote, Bruno Hideo Saiki Silva, Paulo Ramos David João

**Affiliations:** a Hospital Pequeno Príncipe (HPP), Curitiba, PR, Brasil.

**Keywords:** Heart transplantation, Cardiomegaly, Heart neoplasms, Fibroma, Transplante de coração, Cardiomegalia, Neoplasias cardíacas, Fibroma

## Abstract

**Objective:**

Pediatric cardiac tumors are rare and, among them, 90% are benign. Cardiac fibroma is the second most frequent tumor, after rhabdomyoma. The objective of this study is to report a case of cardiac fibroma diagnosed incidentally in a patient admitted with acute viral bronchiolitis.

**Case description:**

A 5-month-old male infant was admitted to the pediatric emergency department with acute viral bronchiolitis requiring hospitalization. He presented a detectable respiratory syncytial virus in oropharyngeal swab, blood test with lymphocytosis and a chest radiography revealed cardiomegaly. Further cardiologic testing was performed detecting elevation of cardiac biomarkers, an electrocardiogram with alteration of left ventricular repolarization and echocardiogram with a heterogeneous mass in the left ventricular, with areas of calcification. A chest angiotomography suggested rhabdomyosarcoma or cardiac fibroma and a magnetic resonance showed a mass, with characteristics suggesting fibroma. The final diagnosis was made after two cardiac catheterizations for biopsy of the lesion, confirming cardiac fibroma by anatomopathological examination. Because the patient had moderate to severe systolic dysfunction, he was submitted to heart transplant.

**Comments:**

One third of cardiac fibromas are asymptomatic, generally diagnosed late through tests ordered for other reasons. The gold-standard test for definitive diagnosis is biopsy. Cardiac fibroma usually does not present spontaneous regression and, in most cases, partial or total surgical resection is necessary. When tumors are unresectable, heart transplantation should be indicated. It is essential to have detailed characterization of the cardiac mass to establish the most appropriate therapeutic approach for each patient.

## RESUMO

**Objetivo:**

Tumores cardíacos pediátricos são raros e, entre eles, 90% são benignos. O fibroma cardíaco é o segundo mais frequente, após o rabdomioma. O objetivo deste trabalho é relatar um caso de fibroma cardíaco, cujo diagnóstico foi incidental em um paciente internado por bronquiolite viral aguda.

**Descrição do caso:**

Lactente do sexo masculino com cinco meses foi atendido em um pronto atendimento pediátrico com quadro de bronquiolite viral aguda, necessitando de hospitalização. Em exames complementares, apresentou vírus sincicial respiratório detectável, hemograma com linfocitose, além de radiografia de tórax com cardiomegalia. Em investigação cardiológica, as enzimas cardíacas encontravam-se elevadas, o eletrocardiograma apresentava alteração de repolarização de parede lateral de ventrículo esquerdo e o ecocardiograma demonstrou massa heterogênea em ventrículo esquerdo, com áreas de calcificação. A angiotomografia de tórax sugeriu rabdomiossarcoma ou fibroma cardíaco e a ressonância magnética demonstrou massa cuja localização e características sugeriram fibroma. O diagnóstico foi conclusivo após dois cateterismos para biópsia da lesão, confirmando fibroma cardíaco pelo anatomopatológico. Em razão da disfunção sistólica moderada a grave, o paciente foi submetido a transplante cardíaco.

**Comentários:**

Um terço dos fibromas cardíacos é assintomático, geralmente diagnosticado tardiamente por meio de exames solicitados por outro motivo. O padrão-ouro para o diagnóstico definitivo é a biópsia. O fibroma cardíaco não costuma apresentar regressão espontânea, sendo submetido, na maioria dos casos, a ressecção cirúrgica parcial ou total. Nos tumores irressecáveis, o transplante cardíaco deve ser indicado. A caracterização detalhada da massa cardíaca é fundamental para traçar a conduta terapêutica mais adequada para cada paciente.

## INTRODUCTION

Heart tumors are rare in children. Among them most cases are benign and only 10% are malignant masses. In recent years, an increase in the diagnosis of these tumors has been noticed, which can be explained by the advancement of non-invasive imaging tests. The initial test to evaluate cardiac masses is echocardiography. After the initial test, computed tomography (CT) scan and cardiac magnetic resonance imaging (MRI) are necessary to detail the mass.^1^

Tumors are differentiated according to histology, location and clinical manifestations.^2^ Rhabdomyoma and fibroma are the most frequent histological types.^3^ In the neonatal period, the most common tumors are teratoma and rhabdomyoma; around 0.14% of cases being diagnosed in utero.^1^

Fibroma, the second most common benign primary cardiac tumor — after rhabdomyoma —, when symptomatic, may present with arrhythmias, heart failure and sudden death.^1^ However, about a third of patients with cardiac fibroma are asymptomatic and the diagnosis is incidental, when imaging tests are requested for other reasons.^4^

The objective of this study is to report a case of cardiac fibroma incidentally diagnosed in an infant with acute viral bronchiolitis (AVB) and to discuss the diagnosis, clinical features and treatment of pediatric cardiac tumors, in particular, cardiac fibroma.

## CASE REPORT

A 5-month-old male infant was admitted to the pediatric emergency department with a history of cough and nasal obstruction for three days. He had a worsening of his general condition in the previous 24 hours, with fever, respiratory effort, reduced appetite and vomiting after feedings.

He was a previously healthy infant, with a gestational history of a low-risk pregnancy, with late-onset prenatal care (3^rd^ trimester), normal obstetric ultrasonography (at 26 weeks of gestational age); however, morphological ultrasonography was not performed. He was born by vaginal delivery, at full-term, with adequate weight for gestational age, without complications.

On physical examination at admission, he was afebrile (36.8°C), tachycardic (158 bpm); tachypneic (50 bpm); with hypoxemia (SpO_2_ 92%). Pulmonary auscultation showed reduced breath sounds on the left hemithorax and the presence of fine rales, rhonchi and diffuse wheezing bilaterally. Hyaline coryza and respiratory distress were observed, with moderate to severe intercostal, subcostal and sternal retraction. Cardiac auscultation showed muffled heart sounds. There was an improvement in oxygen saturation (98%) and respiratory effort with the administration of oxygen by a nasal catheter.

The patient was diagnosed with AVB and admitted to the pediatric ward due to the need of supplemental oxygen and moderate to severe respiratory effort. Laboratory and imaging tests were requested.

**Figure 1:**
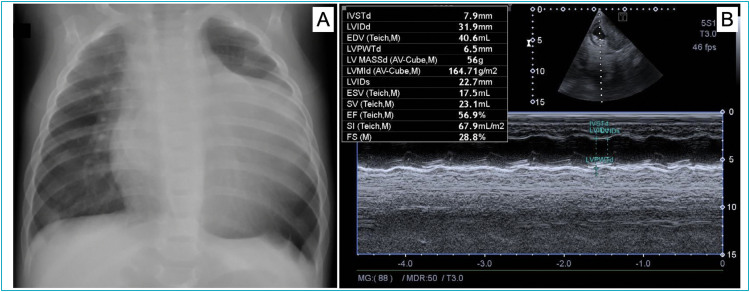
Chest radiography in posteroanterior view (A) demonstrating enlargement of the cardiac silhouette. Echocardiogram (B) showing a heterogeneous mass.

Respiratory syncytial virus was detected on nasopharyngeal swab. Complete blood count revealed normal leukocyte count (8,000/μL), with a predominance of lymphocytes (72%=5,760/μL) and the presence of reactive lymphocytes (2%=160/μL), in addition to mild thrombocytosis (487,000/μL). C-reactive protein (CRP) was normal (5.8 mg/L; reference value: less than 10 mg/dL). Chest radiography showed an enlargement of the cardiac silhouette (Figure 1).

Due to the radiographic finding, cardiac investigation was performed, identifying troponin levels of 86.5 pg/mL (reference value: up to 19.8 pg/mL) and creatine phosphokinase-MB mass (CKMB mass) levels of 18 U/L (reference value: up to 16U/L). Electrocardiogram showed alteration of left ventricular (LV) repolarization. Echocardiogram showed a heterogeneous mass, measuring 52×38 mm, on the lateral wall of the LV, with areas of calcification, preserving the LV inflow and outflow tract (Figure 1).

**Figure 2:**
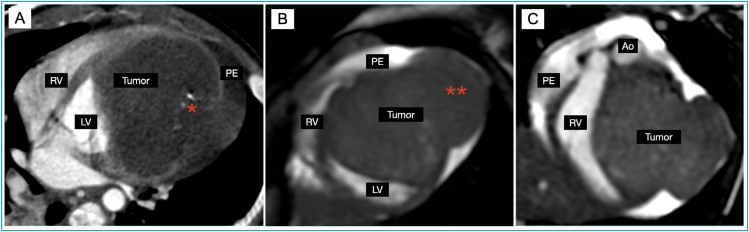
Cardiac angiotomography (A) and cardiac magnetic resonance imaging (B and C), in the 4-chamber (A and B) and 2-chamber short axis (C) views, demonstrating a large expansive lesion in the apicobasal anterolateral wall of the left ventricle (LV), with reduction of left ventricular volume, small foci of central calcifications (*in A), extracardiac extension (**in C) and pericardial effusion (PE in A-C).

Chest CT angiography showed a large intramural cardiac mass in the LV with significant ventricular volume restriction and extracardiac extension, small foci of central calcification and small pericardial effusion (Figure 2).

Cardiac MRI showed a mass in the LV, measuring 5.5×5.3 cm, mobile with the cardiac cycle, extending closely to the pulmonary artery and into the mediastinum. A large mass effect was noted in the LV, with reduction of the ventricular cavity, moderately impairing its global function (LV Ejection Fraction [EF]=46%). T2-weighted images showed a mild myocardial hypersignal and markedly delayed contrast uptake (>10 minutes) (Figure 2).

The extracardiac extension of the lesion and pericardial effusion suggested aggressive behavior. However, the site and tissue characterization, i.e. foci of calcification and markedly slowed enhancement, suggested fibrotic components. Therefore, the hypotheses suggested by the imaging tests were rhabdomyosarcoma and cardiac fibroma.

A Holter was performed and showed a pattern of complete right bundle branch block and isolated supraventricular extrasystoles. A total abdominal MRI and transfontanellar ultrasound were performed, due to the possibility of cardiac rhabdomyosarcoma, both being normal.

The patient underwent cardiac catheterization for biopsy, and fragments were sent for histopathological and immunohistochemical analysis. Since the biopsy was inconclusive, it was decided to start an off-label use of sirolimus.

During the use of sirolimus, periodic echocardiograms were performed, in addition to weekly laboratory tests, including blood count, CRP, serum sirolimus dosage, renal function, liver function, and lipid profile. During this period, serum sirolimus was maintained within the therapeutic range, between 5 and 10 ng/mL, with no changes in other laboratory tests.

The echocardiogram in the second week of sirolimus use identified significant pericardial effusion, causing right ventricle (RV) dysfunction, and a pericardiocentesis was performed, draining 48 mL of serosanguineous fluid.

After three weeks of sirolimus use, a control MRI was performed and there were no significant changes in the size or characteristics of the tumor mass, therefore the off-label use of sirolimus was discontinued.

A new cardiac catheterization with biopsy was performed. Two tissue fragments were sent for anatomopathological analysis, the largest measuring 1.5 cm in length and 0.1 cm in diameter. Histopathological analysis this time was conclusive for cardiac fibroma.

The mass was a histologically benign tumor, but with aggressive behavior due to its size, location and infiltration in the myocardium. The patient required prolonged hospitalization, with the use of vasoactive drugs due to hemodynamic compromise. The tumor was not responsive to the off-label use of sirolimus, requiring cardiological polypharmacy, multiple hospitalizations due to cardiac decompensation, and the patient evolved with functional class 3 congestive heart failure (CHF), reaching an EF of 35%.

Due to the progressive worsening of the EF and, consequently, of the functional class of CHF, it was decided to include the patient in the heart transplantation list, since the tumor resection was impossible due to its infiltration in the left ventricular myocardial wall.

**Figure 3:**
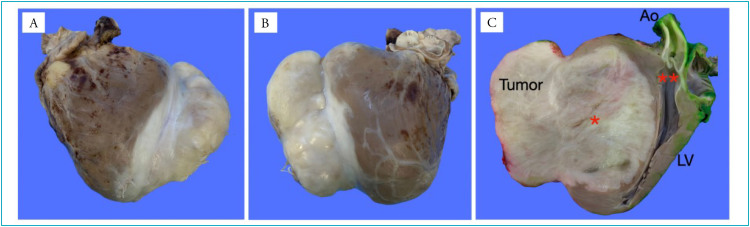
Macroscopic findings, on the anterior surface (A), posterior surface (B) and cross-sectional cut (C), demonstrating a single, solid, non-encapsulated, white, firm, fibrous tumor involving the left ventricle (LV), reaching its anterior wall (*), but preserving the aortic valve (**) and aorta (Ao). There is absence of necrosis or hemorrhage.

**Figure 4:**
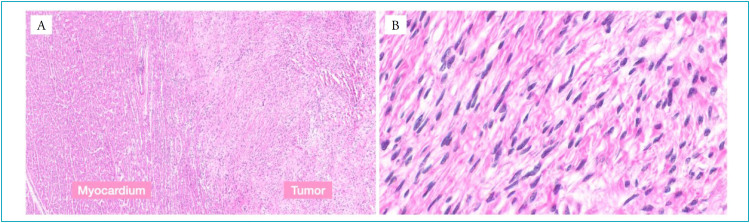
Histopathological findings in hematoxylin-eosin (HE) reveal spindle cell neoplasm of the heart, with infiltrative growth pattern at the margins (HE, 10x) (A). Spindle cells have no atypia, and are arranged in a collagenous background, without evidence of mitosis (HE, 40x) (B).

Six months after diagnosis, the patient underwent heart transplantation at the same hospital where the diagnosis was made, with a good postoperative course. Anatomy and histopathological findings of the surgical specimen confirmed the diagnosis of cardiac fibroma (Figures 3 and 4).

## DISCUSSION

The epidemiology of primary cardiac tumors in the pediatric population is uncertain. Penha et al. estimated that prevalence ranges from 0.0017% and 0.19% and emphasized the increased number of cases diagnosed in recent years due to advances in imaging tests.^1^

Approximately 90% of cardiac tumors are considered benign and, among them, the most frequent histological type is rhabdomyoma, which represents 60% of benign tumors in the first 12 months of life. This percentage decreases to 41% between one and 15 years of age.^2^ Fibroma represents 14% of cases of benign cardiac tumors, occupying the second place of prevalence according to histological type, most of them being diagnosed in children under one year of age.^2,3^

Fibromas originate from fibroblasts of the connective tissue and usually present as single lesions, with variable dimensions. They are most commonly localized in the anterior wall of the LV and interventricular septum, with growth towards the heart chamber.^4,5^ Central calcifications are common findings of fibromas, probably due to reduced blood supply of the tumor.^1,2^ In the present case, the tumor had small foci of central calcification.

Approximately one third of pediatric patients with cardiac fibroma are asymptomatic. Therefore, delayed diagnosis is common, through tests ordered for some other reason.^4^ Signs and symptoms depend on the location and size of the tumor and may include arrhythmias, embolization, heart failure and sudden death.^1,5^ Cardiomegaly is the most frequent imaging finding in these cases. In the presented case, the infant described was hospitalized for AVB and the identification of cardiomegaly on the chest X-ray called attention to initiating the investigation of a possible cardiac mass.

The diagnostic tests of choice are echocardiography, computed tomography and magnetic resonance imaging. Penha et al. point out that, after the advent of the two-dimensional echocardiogram and the greater experience of professionals qualified to perform the tests, there was a great advance in the earlier diagnosis of cardiac tumors. Today, angiography is recommended when there is suspicion of coronary involvement or uncertainty in the diagnosis.^1^ On the other hand, magnetic resonance imaging has a complementary role in providing morphological details of some tumors as well as in assessing myocardial and extracardiac extension and pericardial involvement.^2^

The gold-standard test for definitive diagnosis is biopsy. However, it should be used in more severe cases due to the high risk and invasiveness of the procedure.^6^ In the present case, the definitive diagnosis was made through biopsy, which had to be performed twice to confirm the histological type of the tumor.

Imaging tests suggested rhabdomyosarcoma or fibroma, and the patient underwent a first biopsy which provided inconclusive findings. Due to the risk of a new procedure, it was decided to start an off-label use of sirolimus. Sirolimus, an mTOR inhibitor, has been tested in a great variety of clinical conditions due to its immunosuppressive and antiproliferative effect, often achieving excellent results. Despite such positive evidence, the on-label indications for these rapalogs are still very restricted, especially in children.^7^

As there was no response to the treatment, the off-label use of sirolimus was discontinued, and the patient underwent a second biopsy, which confirmed the diagnosis of cardiac fibroma. Usually, cardiac fibromas do not regress spontaneously and surgical resection is necessary when there is significant obstruction of the inflow or outflow tract of the ventricles, intractable arrhythmia or evidence of embolization. In cases where there is a large cardiac mass, even if the patient is asymptomatic, surgical resection may also be indicated.^4^ Conservative management for this histological type of tumor is controversial.^1^

The surgical options for the treatment of cardiac fibromas include partial and total resection or heart transplantation. Becker reported two cases of patients who underwent partial tumor resection and were clinically well.^5^ The first was a 6-month-old patient who had fibroma in the interventricular septum and there was no further tumor growth after 14 months of the procedure. The second patient was a 22-month-old child with fibroma in the inferior wall of the LV and, after seven years of partial resection, there was no increase in cardiac mass. The main goal of treatment is not the complete resection of the tumor, but the improvement of long-term cardiac function.^5,8^

If the tumor extends through the myocardium, compromising a large cardiac area, and resection is not possible, or causes ventricular arrhythmias or heart failure, heart transplantation should be indicated.^5,9^ This treatment modality should always be kept in mind, but as a last option, reserved for unresectable tumors, as it presents a higher risk of death.^8^ According to the case reported by Rodriguez-Gonzalez et al., heart transplantation was indicated for an infant with fibroma due to the occurrence of ventricular arrhythmias and risk of sudden death.^9^ In the case reported, due to the extension of the tumor and its infiltration into the myocardium, in addition to moderate to severe systolic dysfunction, heart transplantation was indicated.

The present report describes the incidental diagnosis of a cardiac fibroma in an infant with AVB, and draws attention to other differential diagnoses. Thus, the importance of a thorough physical examination and the correct interpretation of subsequent complementary tests is highlighted. In addition, we emphasize the importance of early diagnosis and detailed characterization of the cardiac mass to outline the most appropriate therapeutic approach for each patient.
